# Strigolactone alleviates the salinity-alkalinity stress of *Malus hupehensis* seedlings

**DOI:** 10.3389/fpls.2022.901782

**Published:** 2022-07-22

**Authors:** Changqing Ma, Chuanjie Bian, Wenjie Liu, Zhijuan Sun, Xiangli Xi, Dianming Guo, Xiaoli Liu, Yike Tian, Caihong Wang, Xiaodong Zheng

**Affiliations:** ^1^College of Horticulture, Qingdao Agricultural University, Qingdao, China; ^2^Engineering Laboratory of Genetic Improvement of Horticultural Crops of Shandong Province, Qingdao, China; ^3^College of Life Science, Qingdao Agricultural University, Qingdao, China

**Keywords:** *Malus hupehensis*, strigolactone, salinity-alkalinity stress, ion homeostasis, oxidative stress

## Abstract

Salinity-alkalinity stress can remarkably affect the growth and yield of apple. Strigolactone (SL) is a class of carotenoid-derived compounds that functions in stress tolerance. However, the effects and mechanism of exogenous SL on the salinity-alkalinity tolerance of apple seedlings remain unclear. Here, we assessed the effect of SL on the salinity-alkalinity stress response of *Malus hupehensis* seedlings. Results showed that treatment with 100 μM exogenous SL analog (GR24) could effectively alleviate salinity-alkalinity stress with higher chlorophyll content and photosynthetic rate than the apple seedlings without GR24 treatment. The mechanism was also explored: First, exogenous GR24 regulated the expression of Na^+^/K^+^ transporter genes and decreased the ratio of Na^+^/K^+^ in the cytoplasm to maintain ion homeostasis. Second, exogenous GR24 increased the enzyme activities of superoxide, peroxidase and catalase, thereby eliminating reactive oxygen species production. Third, exogenous GR24 alleviated the high pH stress by regulating the expression of H^+^-ATPase genes and inducing the production of organic acid. Last, exogenous GR24 application increased endogenous acetic acid, abscisic acid, zeatin riboside, and GA3 contents for co-responding to salinity-alkalinity stress indirectly. This study will provide important theoretical basis for analyzing the mechanism of exogenous GR24 in improving salinity-alkalinity tolerance of apple.

## Introduction

Soil salinization-alkalization is a severe environmental factor that inhibits plant growth and productivity for aggravating soil degradation (Jia et al., 2019). To date, 20% of agricultural land is affected by salinity-alkalinity all over the world, and the trend is constantly expanding (Ye et al., 2019). Apple (*Malus domestica*) is a highly valued and widely cultivated fruit around the world (Ma et al., 2019). Apple trees are sensitive to saline-alkali conditions and negatively affected by soil salinization-alkalization. Thus, strategies for improving the salinity-alkalinity tolerance of apple trees should be explored.

Salinity-alkalinity stress simultaneously induces oxidative, high pH, osmotic, and ionic stress (Guo et al., 2017; Jin et al., 2019). Reactive oxygen species (ROS) induced by saline-alkali stress, including superoxide anions (O^2–^), hydrogen peroxide (H_2_O_2_), and singlet oxygen, result in oxidative stress that can lead to plant cell membrane permeability increasing and ion leakage, which may impede plant development (Miller et al., 2010; Zhang et al., 2016; Xu et al., 2021). High pH affects the availability of mineral elements and the absorption of inorganic anions, thus disrupting intracellular ion balance (Yang et al., 2009). Osmotic stress reduces the stomatal openings and decreases plant photosynthesis (Zhang et al., 2019). Moreover, the uptake of K, Mg, and Zn in apple leaves is inhibited, whereas the absorption of Fe, Cu, or Mn is increased under saline-alkali stress (Jia et al., 2019).

Plants resist external pressure *via* several biochemical reaction mechanisms, redox balance, and complex signal transduction pathways throughout their long-term evolutionary process (Jiang et al., 2016; Xu et al., 2021). Plants regulate the osmotic potential by increasing the concentrations of proline, soluble protein, and soluble sugar. Furthermore, multiple elements such as Ca, K, and Fe are involved in photosynthesis, carbon assimilation, and signal transduction in plants (Yang and Guo, 2018). Maintaining a low Na^+^/K^+^ ratio is an important mechanism for preventing cellular damage and nutrient deficiency in plant (Zhang et al., 2018). In addition, plant hormones, such as auxin (IAA), jasmonic acid (JA), cytokinin, and gibberellin (GA) are important for regulating plant development and tolerance to diverse stresses (Zwack and Rashotte, 2015). The application of plant growth regulators can effectively improve plant salt tolerance (Shahzad et al., 2018; Jiang et al., 2021). Some metabolites such as spermidine and γ-aminobutyric acid improve plant salinity-alkalinity tolerance by scavenging ROS and regulating cellular osmotic pressure (Li et al., 2015; Jin et al., 2019). Plants can activate the antioxidant enzyme activities, such as superoxide dismutase (SOD), peroxidase (POD), and catalase (CAT) cooperate together to scavenge ROS and protect plants from oxidative harm (Zheng et al., 2020).

Plant hormones play major roles in regulating plant growth and tolerance to abiotic stress. Strigolactone (SL), as a class of carotenoid-derived compounds, is essential in regulating numerous aspects of plant development (Duan et al., 2019). The enzymes involved in the SL signaling pathway include ubiquitin-related protein F-box leucine-rich repeat protein (D3/MAX2), SL receptor α/β hydrolyzyme (D14), and transcriptional repressor Clp ATPase family protein (D53/SMXL6/7/8) (Yao et al., 2016; Shabek et al., 2018). The exogenous application of GR24, a synthesized SL, significantly increases the enzymatic activities of SOD and POD, decreases the malondialdehyde (MDA) content, and mitigate the adverse effects of salt stress in rice (Ling et al., 2020). Moreover, exogenous GR24 application protects the chlorophyll and maintains the photosynthetic rate of apple seedlings under KCl stress (Zheng et al., 2020). In addition, exogenous GR24 can improve the cold and drought resistance of rape seedlings by improving cell viability and inhibiting the production of reactive oxygen species (Zhang X. et al., 2020; Wang et al., 2021). Therefore, we hypothesized that SL might play positive roles on salinity-alkalinity stress in apple seedlings. However, the mechanisms and functions of GR24 under salinity-alkalinity stress in apple remain unknown.

In the present study, we explored the functions of exogenous GR24 in *Malus hupehensis*, one of the important rootstocks in apple culture, under salinity-alkalinity stress. Different concentrations of exogenous GR24 were applied on *M. hupehensis* seedlings under salinity-alkalinity stress, the positive regulation of GR24 was evaluated in terms of the photosynthetic system, oxidative damage, osmotic balance, and ion homeostasis. The expression levels of ion transporter genes, key SL signaling pathway genes, and SL biosynthesis genes under GR24 treatments were also determined. This study helped clarify the regulatory mechanism of SL in apple plants under salinity-alkalinity stress and provided a new way to improve salinity-alkalinity tolerance in apple production.

## Materials and methods

### Plant materials and growth conditions

After low-temperature vernalization, the seeds of *M. hupehensis* (an apple rootstock with apomixis characteristics) were sown in nutrient soil and grown in a greenhouse under controlled temperature (25 ± 2°C), photoperiod (16/8 h day/night), humidity (60–65%), and light intensity (100 μmol/m^2^/s). After one-month-old, when the seedlings developed into to four leaves, they were transplanted into a plastic pot and irrigated with Hoagland solution every 3 days. Ten days later, seedlings with similar growth status were selected for subsequent saline-alkali stress and exogenous GR24 treatment.

### Saline-alkali stress and exogenous GR24 treatment assay

A total of 200 *M. hupehensis* seedlings were randomly divided into five groups. The seedlings in group I were watered with a complete nutrient solution as the control, group II were treated by 100 mM NaHCO_3_ and NaCl with concentration ratio of 1:1. On the basic of group II, groups III-V were treated with the 10, 100, and 1,000 μM of exogenous GR24, respectively. GR24 (Solarbio, Beijing, China) was sprayed every 3 days. After 15 days of treatment, the seedlings were photographed, and the wilting rate, fresh weight, and dry weight were measured. Both the technical and biological duplications of each experiment were repeated thrice.

### Measurement of chlorophyll content, photosynthetic parameters, and root activity

After 15 days of saline-alkali stress and exogenous GR24 treatment, 20 apple seedlings from each group were randomly selected to determine the chlorophyll content and basic photosynthetic parameters. Four leaves of each seedlings were measured. Under light condition, the chlorophyll content was measured using SPAD-502 Plus (Konica Minolta, Tokyo, Japan). The photosynthesis rate, transpiration rate, and stomatal conductance were measured using the CIRAS-3 portable photosynthetic system (PP Systems, Amesbury, United States). The light intensity was controlled at 800 μmol/m^2^/s at an approximately 50% humidity, and the temperature was set at 22°C. 2,3,5-triphenyltetrazolium chloride (TTC) method was applied for qualitatively and quantitatively assess the root activity according to Gong et al. (2017). Each experiment was repeated thrice.

### Determination of reactive oxygen species levels and malondialdehyde content

Nitroblue tetrazolium and 3,3-diaminobenzidine were used to stain H_2_O_2_ and O^2–^, respectively. The H_2_O_2_ level was measured using H_2_O_2_ kits (Grace, Suzhou, China). The MDA content of the leaves was measured using a plant MDA extraction kit (Grace, Suzhou, China). Three biological duplications for each experiment were set.

### Determination of antioxidant enzyme activity and organic acid content

Fresh leaves (0.5 g) were ground in 5 ml of extracted buffer after saline-alkali and exogenous GR24 treatment for 15 days. After centrifugation at 12,000 rpm for 10 min, the supernatants were immediately used for SOD, POD, and CAT content assay. SOD, POD, and CAT kits (Grace, Suzhou, China) were used to detect the activities of antioxidant enzymes according to the manufacturer’s instructions. The malic acid and citric acid content of apple leaves were measured using Malic acid assay Kit and Citric acid assay Kit (Suzhou Geruisi Biotechnology, Suzhou, china), respectively. Each experiment was repeated thrice.

### Determination of electrolyte leakage and osmolyte content

After saline-alkali and exogenous GR24 treatment for 15 days, fresh leaves (0.5 g) from each group were used for the detection of electrolyte leakage and osmolytes. Electrolyte leakage was measured as described by Ahmad et al. (2016). Osmolytes including proline, soluble sugar, and soluble protein were detected. Proline content was measured as described by Wani et al. (2017). Soluble sugar and soluble protein contents were determined as described by Sharma et al. (2019) and Qiu et al. (2019) respectively. Each experiment was repeated thrice.

### Quantification of mineral elements assay

The apple seedlings were collected and washed with deionized water to remove the excess impurities after 15 days of saline-alkali stress and GR24 treatment. The leaves were dehydrated at 105°C for 30 min and baked at 80°C for 72 h. Afterward, 0.5 g of kiln-dried leaves was ground into powder and added with 12 ml of HNO_3_ and HClO_4_ with the ratio of 5:1. After digestion, the solution was diluted with deionized water to 25 ml. The concentrations of sodium (Na), potassium (K), calcium (Ca), iron (Fe), magnesium (Mg), and phosphorus (P) were determined through inductively coupled plasma-optical emission spectrometry (PerkinElmer, Waltham, United States) as described by Su et al. (2020).

### Measurement of endogenous hormone

After saline-alkali and exogenous GR24 treatment for 15 days, the endogenous acetic acid (IAA), gibberellin3 (GA3), zeatin riboside (ZR), and jasmonic acid (JA) concentrations were determined. Fresh leaves (0.5 g) were prepared for phytohormone extractions, and hormonal analysis and quantification were performed *via* electrospray ionization-high-performance liquid chromatography-tandem mass spectrometry, as described by Min et al. (2018). Each experiment was repeated thrice.

### Real-time quantitative PCR assay

Total RNA was extracted from each group by using the RNA prep pure Plant Plus kit (Tiangen, Beijing, China), which includes RNase-free DNase treatment. Total RNA was adjusted to the same concentration for cDNA synthesis by using 5 × All-In-One RT MasterMix (ABM, Sydney, Australia) according to the manufacturer’s instructions. LightCycler^®^ 480 II system (Roche, Rotkreuz, Switzerland) was used for the qPCR assay, and the primers are listed in Supplementary Table 1. The *M. hupehensis* actin gene (GenBank accession number GQ339778.1) was used to normalize gene expression levels. Data were analyzed using the 2^–ΔΔCt^ method (Min et al., 2018). All qRT-PCR experiments were repeated thrice.

### Statistical analysis

Data were subjected to ANOVA followed by Fisher’s LSD or Student’s *t*-test analysis. Statistically significant differences were indicated by *P* < 0.05. Statistical computations were conducted by using SPSS software (IBM, Armonk, NY, United States).

## Results

### Effects of exogenous GR24 on the growth of apple seedlings under saline-alkali stress

**FIGURE 1:**
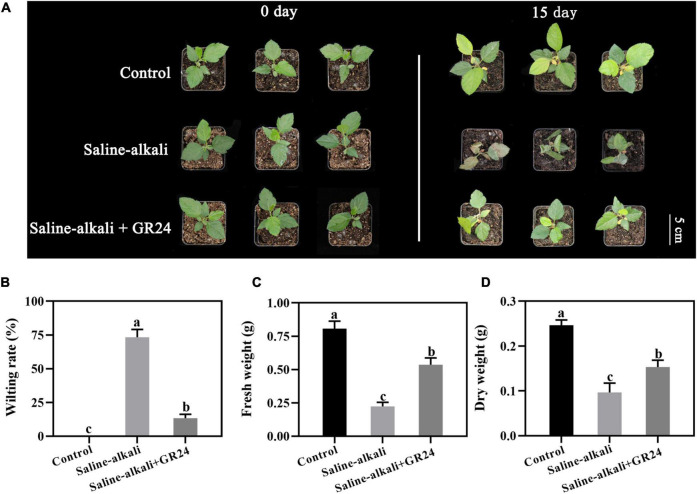
Phenotypes of *Malus hupehensis* seedlings treated with salinity-alkalinity stress and exogenous 100 μM GR24 on day 0 and day 15 **(A)**. Effect of GR24 on wilting rate **(B)**, fresh weight **(C)**, and dry weight **(D)** of apple seedlings after salinity-alkalinity stress for 15 days. The bar **(A)** represents 4.0 cm. The data represent the mean ± SD of three biological replicates. Different lowercase letters indicate significant differences according to Fisher’s least significant difference (*P* < 0.05).

The apple seedlings were seriously damaged by saline-alkali stress, and the leaves became withered and chlorotic after 15 days. After the treatment of low (10 μM) and high (1 mM) concentrations of GR24, the growth vigor of the seedlings was much better than those without GR24 treatment, but the leaves remained withered and chlorotic. At the low (10 μM) and the high (1 mM) concentrations, the wilting rates of the seedlings substantially decreased from 73.3% to 45% and 48.3%, respectively (Supplementary Figure 1B), and the fresh weights remarkably increased to 0.34 and 0.36 g, respectively (Supplementary Figure 1C). However, when the middle concentration of GR24 (100 μM) was applied, the growth vigor of the seedlings under saline-alkali stress was similar to that of the control under normal conditions, and the wilting rate of the seedlings remarkably decreased to 13.3% compared with those without GR24 treatment under saline-alkali stress (Supplementary Figure 1B). In addition, under saline-alkali stress, the fresh and dry weights of the seedlings sprayed with 100 μM GR24 increased significantly compared with that without exogenous GR24 (Figures 1C,D). The result suggested that exogenous GR24 could protect the apple seedlings from saline-alkali stress, and the concentration of 100 μM GR24 exhibited the best effect, which was therefore selected for further research.

### Effects of exogenous GR24 on the chlorophyll content and photosynthetic parameters under saline-alkali stress

**FIGURE 2:**
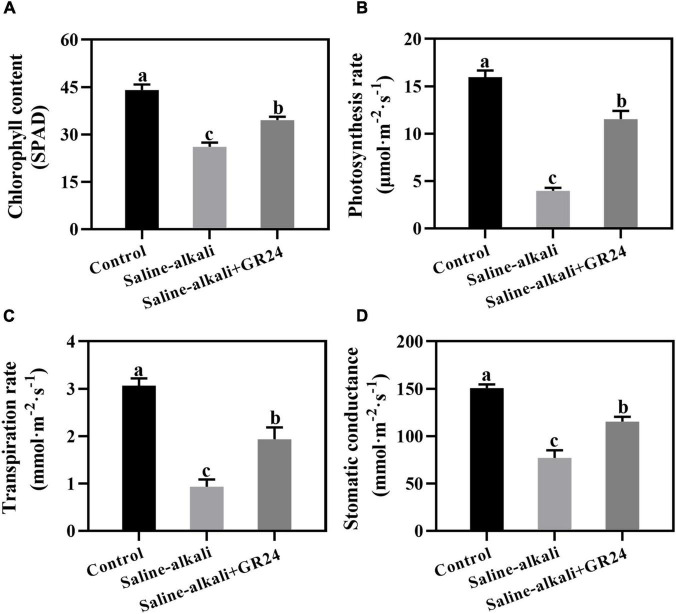
Effects of exogenous GR24 application on the chlorophyll content **(A)**, photosynthetic rate **(B)**, transpiration rate **(C)** and stomatic conductance **(D)** of *Malus hupehensis* seedlings after salinity-alkalinity stress for 15 days. The data represent the mean ± SD of three biological replicates. Different lowercase letters indicate significant differences according to Fisher’s least significant difference (*P* < 0.05).

Exogenous GR24 could prevent the chlorosis of the apple seedlings under saline-alkali stress (Figure 1). To explore the physiological mechanism, we determined the chlorophyll content and photosynthetic parameters after saline-alkali stress and GR24 treatment for 15 days. The chlorophyll content of the apple seedlings sharply decreased from 44.0 SPAD to 26.1 SPAD under saline-alkali stress. When exogenous GR24 was applied, the chlorophyll content of apple seedlings under saline-alkali stress remarkably increased to 34.5 SPAD (Figure 2A). The photosynthetic parameters, including photosynthesis rate, transpiration rate, and stomatic conductance, under saline-alkali stress and exogenous GR24 treatment followed a similar variation tendency as the chlorophyll content. All values were substantially inhibited under saline-alkali stress but increased after exogenous GR24 application (Figures 2B–D), especially the photosynthesis rate. Under saline-alkali stress, the photosynthesis rate decreased significantly from 16 μmol/m^2^/s to 4 μmol/m^2^/s but recovered to 11.5 μmol/m^2^/s when exogenous GR24 was applied (Figure 2B). Therefore, exogenous GR24 could protect the chlorophyll level and photosynthetic system against saline-alkali stress.

### Effects of exogenous GR24 on the oxidative damage and antioxidant enzyme activity of apple seedlings under saline-alkali stress

**FIGURE 3:**
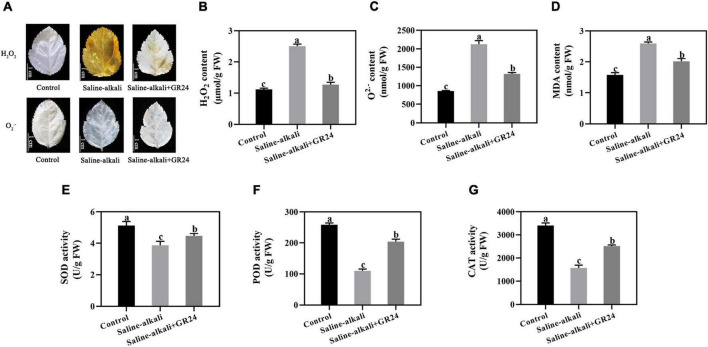
Effects of GR24 treatment on H_2_O_2_, O^2–^ **(A)**, and H_2_O_2_ content **(B)**, O^2⋅–^ content **(C)**, malondialdehyde (MDA) content **(D)**, superoxide dismutase (SOD) activity **(E)**, peroxidase (POD) activity **(F)**, and catalase (CAT) activity **(G)** under salinity-alkalinity stress. The bar **(A)** represents 1.0 cm. The data represent the mean ± SD of three biological replicates. Different lowercase letters indicate significant differences according to Fisher’s least significant difference (*P* < 0.05).

Plants produce ROS under stress conditions. The staining results of superoxide (O^2–^) and H_2_O_2_ revealed that the leaves of apple seedlings were seriously damaged by saline-alkali stress (Figure 3A). When exogenous GR24 was sprayed, the O^2–^ and H_2_O_2_ levels remarkably decreased (Figures 3A–C). The variation tendency of the MDA content was similar to that of O^2–^ and H_2_O_2_. The MDA content under saline-alkali stress (2.6 nmol/g) was more than 1.6 times that of the control group (1.6 nmol/g), but was significantly decreased to 2 nmol/g after exogenous GR24 was applied (Figure 3D).

The activities of antioxidant enzymes were also measured. Under saline-alkali stress, the SOD activity decreased from 5.1 U/g to 3.8 U/g, but recovered to 4.4 U/g after exogenous GR24 was applied (Figure 3E). The POD activity under saline-alkali stress significantly decreased from 259.0 U/g to 110.0 U/g. However, when exogenous GR24 was applied, the POD activity recovered to 203.7 U/g (Figure 3F). CAT activity was only 1572.0 U/g under saline-alkali stress, while increased to 2515.7 U/g when exogenous GR24 was applied (Figure 3G).

### Effects of exogenous GR24 application on the electrolyte leakage and osmolytes under saline-alkali stress

**FIGURE 4:**
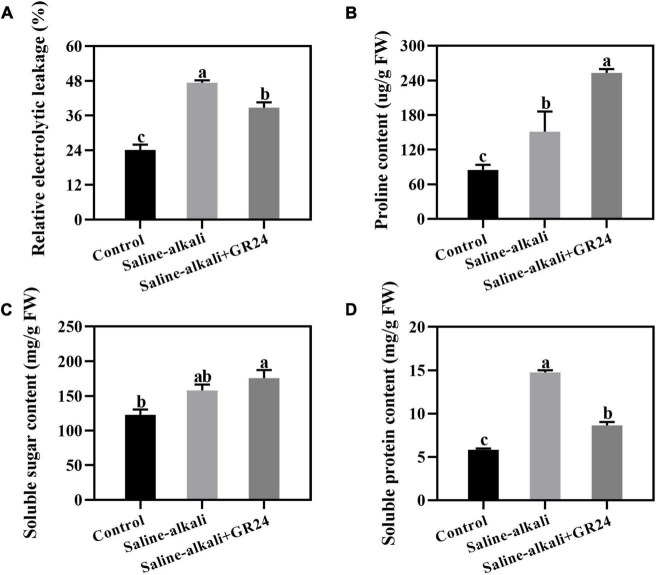
Effects of exogenous GR24 treatment on electrolyte leakage **(A)**, proline content **(B)**, soluble sugar content **(C)**, and soluble protein content **(D)** under salinity-alkalinity stress. The data represent the mean ± SD of three biological replicates. Different lowercase letters indicate significant differences according to Fisher’s least significant difference (*P* < 0.05).

Electrolyte leakage was detected after saline-alkali stress and exogenous GR24 treatment for 15 days. After saline-alkali stress, the electrolyte leakage remarkably increased from 24.1% to 47.4% but decreased to 38.7% when exogenous GR24 was applied (Figure 4A). Osmolyte content under saline-alkali stress and exogenous GR24 treatment was also detected. The proline, soluble sugar, and soluble protein contents increased under saline-alkali stress. When exogenous GR24 was applied, proline content notably increased from 151.2 μg/g to 253.0 μg/g (Figure 4B), while the soluble sugar content had no significant changes (Figure 4C). However, the soluble protein content substantially decreased from 14.7 mg/g to 8.6 mg/g when exogenous GR24 was applied (Figure 4D).

### Effects of exogenous GR24 on the mineral elements of apple seedlings under saline-alkali stress

**FIGURE 5:**
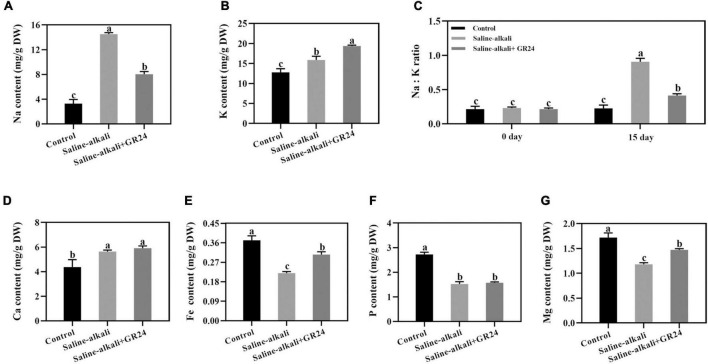
Effects of GR24 treatment on Na content **(A)**, K content **(B)**, and Na^+^/K^+^ ratio **(C)**, Ca content **(D)**, Fe content **(E)**, P content **(F)**, Mg content **(G)** under salinity-alkalinity stress. The data represent the mean ± SD of three biological replicates. Different lowercase letters indicate significant differences according to Fisher’s least significant difference (*P* < 0.05).

The mineral elements of apple seedlings were measured after saline-alkali stress and exogenous GR24 treatment for 15 days. The Na content was significantly increased from 3.3 mg/g to 14.1 mg/g under saline-alkali stress but decreased to 8.0 mg/g after exogenous GR24 treatment (Figure 5A). When exogenous GR24 was applied, the K level substantially increased from 14.6 mg/g to 15.9 mg/g under saline-alkali stress and increased to 19.4 mg/g (Figure 5B). As an important indicator of plant tolerance to abiotic stress, Na^+^/K^+^ ratio was also detected, the Na^+^/K^+^ ratio notably increased to 90.6% under saline-alkali stress but decreased to 41.4% by exogenous GR24 after 15 days treatment (Figure 5C). The Ca content substantially increased from 4.4 mg/g to 5.6 mg/g under saline-alkali stress but that had no significant changes when exogenous GR24 was applied (Figure 5D). The variation tendencies of Fe and Mg were similar. The Fe and Mg contents of apple seedlings with exogenous GR24 treatment increased to 0.305 mg/g and 1.472 mg/g respectively, compared with that without GR24 treatment under saline-alkali stress (Figures 5E,G). However, the P content did not change substantially under saline-alkali stress and exogenous GR24 treatment (Figure 5F).

### Effects of exogenous GR24 on endogenous hormone content under saline-alkali stress

**FIGURE 6:**
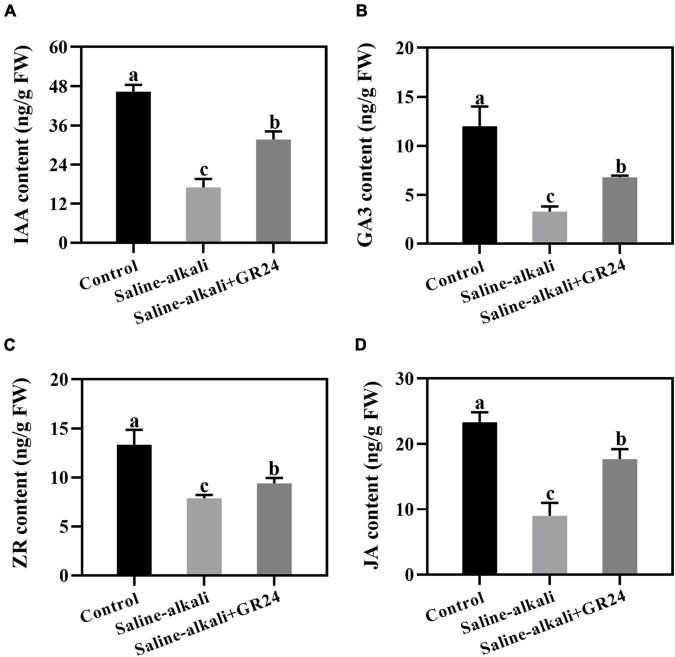
Effects of GR24 treatment on the contents of auixn **(A)**, glbberellin **(B)**, cytokinin **(C)** and jasmonic acid **(D)** under salinity-alkalinity stress. The data represent the mean ± SD of three biological replicates. Different lowercase letters indicate significant differences according to Fisher’s least significant difference (*P* < 0.05).

Plant hormone regulates the mechanisms of plant stress responses. Under saline-alkali stress, the IAA, GA3, ZR, and JA levels notably decreased. When exogenous GR24 was applied, all of them increased substantially (Figures 6A–D). The result indicated that exogenous GR24 increased the sensitivity of endogenous hormone to regulate the tolerance of apple seedlings to saline-alkali stress.

### Effects of exogenous GR24 on the root activity and organic acid contents of apple seedlings under saline-alkali stress

**FIGURE 7:**
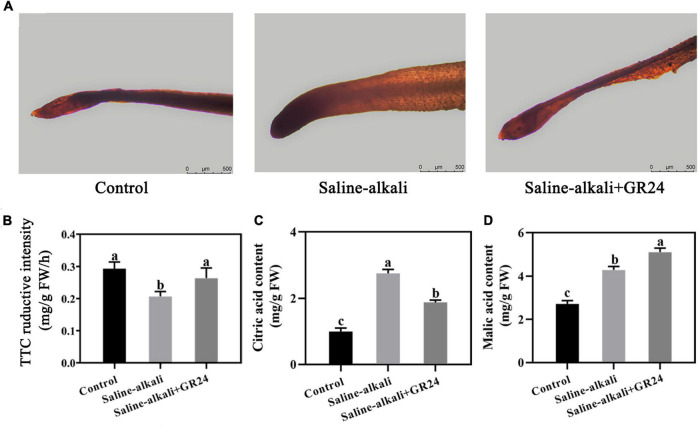
Effects of GR24 treatment on root activity **(A)**, triphenyltetrazolium chloride (TTC) reductive intensity of apple roots **(B)**, citric acid content of apple leaves **(C)**, and malic acid content of apple leaves **(D)** under salinity-alkalinity stress. The data represent the mean ± SD of three biological replicates. Different lowercase letters indicate significant differences according to Fisher’s least significant difference (*P* < 0.05).

Triphenyl tetrazolium chloride is a REDOX compound, which is commonly used as the receptor of H^+^ for the alysis of the activity of different enzymes. Deep red color reported the highest content of H^+^. After 15 days of saline-alkali stress, the root tips of apple seedlings with exogenous GR24 treatment were darker red than those without GR24 treatment under saline-alkali stress (Figure 7A). The TTC reductive intensity in apple roots decreased from 0.29 mg/g FW/h to 0.20 mg/g FW/h under saline-alkali stress. When exogenous GR24 was sprayed, the TTC reductive intensity in apple roots increased to 0.26 mg/g FW/h (Figure 7B). Moreover, the contents of citric and malic acid in apple leaves remarkably increased to 2.76 mg/g and 4.28 mg/g, respectively, under saline-alkali stress. When exogenous GR24 was applied, the citric acid content of plant leaves under saline-alkali stress decreased to 1.88 mg/g, but the malic acid content in leaves of apple seedlings increased to 5.1 mg/g (Figures 7C,D).

### Effects of exogenous GR24 on the expression levels of salinity-alkalinity-related genes in apple seedlings under salinity-alkalinity stress

**FIGURE 8:**
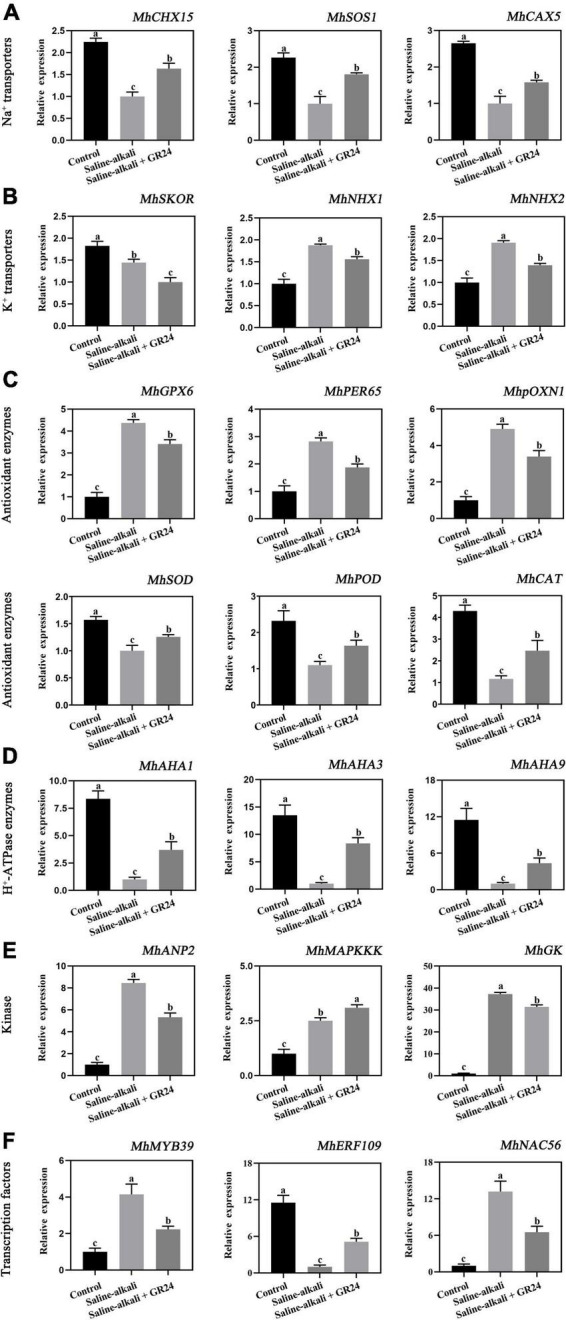
The expression level of the 21 candidate genes which divided into Na^+^ transporters (*MhCHX15*, *MhSOS1*, and *MhCAX5*) **(A)**, K^+^ transporters (*MhSKOR*, *MhNHX1*, and *MhNHX2*) **(B)**, H^+^-ATPase (AHA) enzyme family genes (*MhAHA1*, *MhAHA3*, and *MhAHA9*) **(C)**, antioxidant enzymes (*MhGPX6*, *MhPER65*, *MhpoxN1, MhSOD*, *MhPOD* and *MhCAT*) **(D)**, kinase (*MhANP2*, *MhMAPKKK*, and *MhGK*) **(E)**, and transcription factors (*MhMYB39*, *MhERF019*, and *MhNAC56*) **(F)**. The data represent the mean ± SD of three biological replicates. Different lowercase letters indicate significant differences according to Fisher’s least significant difference (*P* < 0.05).

To elucidate the mechanism of exogenous GR24 involvement in the salinity-alkalinity stress response, we performed qPCR to detect the expression levels of stress-related genes under salinity-alkalinity stress and exogenous GR24 treatment. As shown in Figure 8, exogenous GR24 remarkably upregulated the expression of *MhCHX15*, *MhSOS1*, and *MhCAX5*, as Na^+^ transporter genes, by 1.63, 1.81, and 1.58 times, respectively. The expression of two K^+^ transporter genes, namely, *MhNHX1* and *MhNHX2*, remarkably increased under salinity-alkalinity stress. When exogenous GR24 was applied, the expression levels of them decreased to 1.58 and 1.41 times, respectively, whereas that of *MhSKOR* was substantially downregulated after saline-alkali stress and exogenous GR24 treatment. Moreover, exogenous GR24 significantly upregulated the expression of *MhAHA1*, *MhAHA3*, and *MhAHA9*, as H^+^-ATPase (AHA) enzyme family genes to 2.57, 8.36, and 4.36 times, respectively. The expression levels of antioxidant enzyme genes *MhGPX6*, *MhPER65*, *MhpOXN1*, were significantly induced by salinity-alkalinity stress and were substantially decreased after exogenous GR24 treatment. However, the expression levels of *MhSOD*, *MhPOD* and *MhCAT* were significantly decreased under the salinity-alkalinity stress, while exogenous GR24 significantly upregulated the expression levels of *MhSOD*, *MhPOD* and *MhCAT* to 1.36, 1.93 and 2.47 times, respectively. Moreover, the expression of three kinases, namely, *MhANP2*, *MhMAPKKK*, and *MhGK*, and three selected transcription factors, namely, *MhMYB39*, *MhERF019*, and *MhNAC56*, remarkably changed under salinity-alkalinity stress and exogenous GR24 treatment (Figure 8). This finding indicates their potential important functions in plant response to salinity-alkalinity stress and SL signaling transduction pathway.

### Effects of exogenous GR24 on the expression levels of strigolactones biosynthesis and signal transduction pathway genes in apple seedlings under salinity-alkalinity stress

**FIGURE 9:**
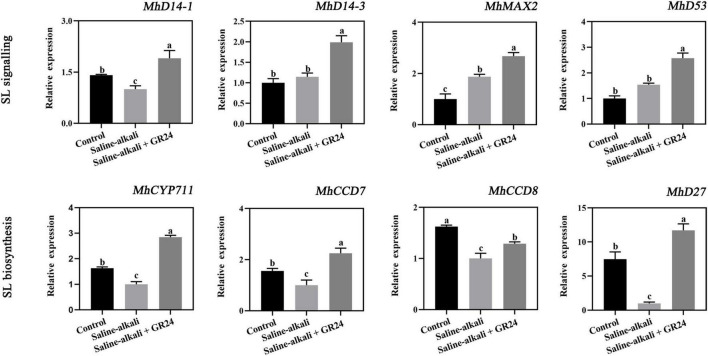
The expression level of the four genes in SL signal transduction pathway (*MhD14-1*, *MhD14-3*, *MhMAX2*, and *MhD53*), and four SL biosynthesis genes (*MhCYP711*, *MhCCD7*, *MhCCD8*, and *MhD27*) under salinity-alkalinity stress and exogenous GR24 treatment for 15 days. The data represent the mean ± SD of three biological replicates. Different lowercase letters indicate significant differences according to Fisher’s least significant difference (*P* < 0.05).

To determine whether the SL biosynthesis and signal transduction pathway genes were involved in the response to salinity-alkalinity stress, we screened out eight genes, which are also involved in the response to salinity-alkalinity stress by analyzing RNA-Seq in apple. The four SL signal transduction pathway genes included a ubiquitin ligase component F-box protein gene (*MhMAX2*) and three DWARF14 genes (*MhD14-1*, *MhD14-2*, and *MhD53*). The expression of *MhD14-1* was decreased by salinity-alkalinity stress. The transcription levels of *MhMAX2* and *MhD53* were increased by salinity-alkalinity stress. However, all of the four genes were induced by exogenous GR24 treatment (Figure 9). The expression of the four SL biosynthetic enzyme genes, including a cytochrome gene (*MhCYP711*), two carotenoid cleavage dixoygenase genes (*MhCCD7* and *MhCCD8*), and a 9-*cis*/all-*trans*-β-carotene isomerase gene (*MhD27*), were also quantified by qPCR. The results showed that the expression levels of these four genes were decreased by salinity-alkalinity stress but remarkably induced by exogenous GR24 treatment (Figure 9).

## Discussion

Salinity-alkalinity stress is an important factor that limits apple production. Damage caused by alkaline salt stress is more severe than that only caused by neutral salt stress. Phytohormones are inherent signaling molecules, which regulate the growth and development of plants by producing complex responses under various stresses (Verma et al., 2016; Waadt et al., 2022). SL, as a group of carotenoid-derived plant hormones, play an important role in regulating various developmental and adaptation processes in plants (Wang et al., 2020). The external application of SL analog GR24 is a promising approach for stablishing various abiotic stress tolerances in plants (Bhoi et al., 2021). Notably, the exogenous application of GR24 could improve plant growth and photosynthesis under salinity and drought stress in plant (Zulfqar et al., 2020; Bidabadi and Sharif, 2021). However, the effect of exogenous GR24 application on plants under salinity-alkalinity stress has not been reported. In the present study, we applied different concentrations of GR24 to salinity-alkalinity-stressed apple seedlings and found that the effects of 100 μM GR24 application was much better than that at 10 μM and 1 mM, and had the lowest wilting rate and the highest fresh weight (Supplementary Figure 1). This study first reported the function of GR24 in apple tolerance to salinity-alkalinity stress.

Chlorophyll is essential for photosynthesis. Saline-alkali stress damages the chlorophyll metabolism and photosynthesis in plant (Hu et al., 2016; Feng et al., 2021). In the present study, salinity-alkalinity stress could significantly inhibit the chlorophyll content and photosynthesis rate in apple (Ding et al., 2010; Guo et al., 2015). It was reported that GR24 treatment displayed greater tolerance to KCl stress by regulating chlorophyll components and photosynthetic rate in apple (Zheng et al., 2020). Our results showed that the application of exogenous GR24 could remarkably increase the stomatic conductance and transpiration rate of apple under salinity-alkalinity stress. Furthermore, the chlorophyll content and photosynthesis rate were much higher in GR24-treated than in non-GR24-treated salinity-alkalinity-stressed apple seedlings (Figure 2). Thus, exogenous GR24 could protect the photosynthetic system from salinity-alkalinity damage.

Plants normally suffer ionic toxicity, high pH, oxidative damage, and osmotic stress from saline-alkaline conditions. The ionic toxicity caused by salinity-alkalinity stress can lead to the excessive accumulation of Na^+^ in the cytoplasm, thereby affecting plant growth (Javid et al., 2012; Xu et al., 2020). In plant responses to salt stress, the ionic toxicity can lead to an imbalance in cytosolic Na^+^/K^+^ ratio and disrupt normal plant growth (Dai et al., 2018; Ma et al., 2021). In the present experiment, the contents of Na^+^ and K^+^ increased under salinity-alkalinity stress (Figure 5). The increase of K^+^ content was attributed to the achievement of balance for Na^+^ and K^+^ homeostasis under salinity-alkalinity stress. When exogenous GR24 was applied to the apple leaves, Na^+^ content decreased with increased K^+^ content, thus decreasing the leaf Na^+^/K^+^ ratio (Figure 5). This condition is similar to the findings of Zulfqar et al. (2020), in which GR24 treatment increased K^+^ content and reduced Na^+^/K^+^ ratio in sunflower (*Helianthus annuus*) under salt stress. Plants have evolved some important protein, which may protect themselves and reduce the poisoning of Na^+^, such as the cation/H^+^ exchangers, salt overly sensitive l (SOS1), and cation exchangers, which expel Na^+^ from cells. Our results showed that *MhCHX15*, *MhSOS1*, and *MhCAX5* expression levels were increased by exogenous GR24 treatment under salinity-alkalinity stress (Figure 8). We assumed that these three genes could function to balance Na^+^ homeostasis in the cytoplasm under salinity-alkalinity stress. Stellar K^+^-outward rectifier (SKOR) is responsible for K^+^ efflux from the cytoplasm to the outside of the cell (Xue et al., 2019). The vacuolar K^+^/H^+^ antiporters (NHX) in the tonoplast facilitate K^+^ influx and efflux in the vacuoles (Xue et al., 2019; Xu et al., 2020). The expression levels of *MhSKOR*, *MhNHX1*, and *MhNHX2* were substantially inhibited after GR24 treatment (Figure 8). Therefore, exogenous GR24 could decrease the expulsion of K^+^ out of the cells to ensure Na^+^/K^+^ homeostasis in the cytoplasm under salinity-alkalinity stress. Moreover, Ca regulates plant signal transduction pathways under salt stress (Hu et al., 2016). Therefore, the Ca content was remarkably induced by exogenous GR24 possibly as the salinity-alkalinity stress response of the apple seedlings to balance Na^+^/Ca^2+^ in the cytoplasm. Fe is essential for plant resistance to oxidative stress (Dai et al., 2018). Therefore, Fe content increases after GR24 treatment in response to oxidative damage caused by salinity-alkalinity stress.

High pH can reduce the availability of mineral elements and affect intracellular ion balance (Palmgren and Nissen, 2011). The plasma membrane (PM) H^+^-ATPase extrudes protons from the plant cell, thus generating an electrochemical gradient across the plasma membrane and plays a pivotal role in abiotic stresses, such as salinity, drought, and temperature (Palmgren and Nissen, 2011; Janicka et al., 2018; Xue et al., 2019). Exogenous GR24 application increases H^+^ and malic acid contents (Figures 7A,D). Moreover, the expression levels of three AHA enzyme family genes, *MhAHA1*, *MhAHA3*, and *MhAHA9*, were increased by exogenous GR24 treatment under salinity-alkalinity stress. Therefore, exogenous GR24 alleviates the high-pH stress of apple seedlings by regulating the expression of H^+^-ATPase genes and inducing the production of organic acid.

Oxidative damage is caused by excessive ROS, which is an important signal molecule that regulates plant metabolism, growth, and stress response (Zheng et al., 2020). The application of GR24 diminishes the H_2_O_2_ and MDA contents in *Triticum aestivum* under drought condition (Sedaghat et al., 2017). In the present experiment, we found that the O^2⋅–^, H_2_O_2_, and MDA contents were remarkably induced by salinity-alkalinity stress, and exogenous GR24 application can decrease their contents (Figure 3), suggesting that SL may act as ROS scavenger and reduce lipid peroxidation in apple seedlings under salinity-alkalinity stress. Enzymatic antioxidant systems include three main antioxidant enzymes, namely, SOD, POD, and CAT (Abdelaal et al., 2018; Min et al., 2018). Our results indicated that salinity-alkalinity stress differentially affects the contents of antioxidant enzymes, the SOD, POD and CAT activities were inhibited under salinity-alkalinity stress. When exogenous GR24 was applied, the SOD, POD and CAT activities substantially increased (Figure 3). This finding was similar with that of exogenous SL treatment under KCl stress (Zheng et al., 2020). Furthermore, six antioxidant enzyme genes (*MhGPX6*, *MhPER65*, *MhpOXN1*, *MhSOD*, *MhPOD* and *MhCAT*) were substantially affected by exogenous GR24, and the tendencies of *MhSOD*, *MhPOD* and *MhCAT* expression levels were correlated with SOD, POD and CAT activities (Figure 8). Thus, exogenous GR24 could alleviate oxidative damage by regulating the expression of antioxidant enzyme genes, enhancing the enzyme activities of SOD, POD, and CAT under salinity-alkalinity stress.

Plants adapt to osmotic stress mainly by regulating the accumulation of osmolytes, such as sugars and amino acids, to reduce cellular osmotic potential and remove excessive ROS (Blumwald, 2003). Our results indicated that electrolyte leakage was remarkably induced by salinity-alkalinity stress but inhibited by exogenous GR24 treatment. This result was consistent with previous findings, in which SL could protect plants from osmotic stress (Wang et al., 2019). To investigate the function mechanism of GR24 on osmotic stress, we detected the soluble sugar, soluble protein, and proline contents under salinity-alkalinity and GR24 treatment. The results indicated that exogenous GR24 could affect the proline content under salinity-alkalinity stress (Figure 4). Exogenous SL could increase proline concentration and alleviate the KCl stress of *M. hupehensis*. Therefore, exogenous GR24 could protect plants from osmotic stress by affecting the accumulation of proline.

Phytohormones mediate various environmental stresses and thus regulate plant growth adaptation (Yu et al., 2020). The involvement of these hormones in plant salinity-alkalinity tolerance and the interactions among them remain to be elucidated. In our study, GR24 was sprayed to the apple leaves. However, the apple roots also exhibited better root activity under saline-alkali stress (Figure 7). Since applying with exogenous GR24 significantly improved the IAA content in apple leaves (Figure 6), the good activity phenotype of apple roots might caused by the systemic regulation of IAA. Haim et al. (2021) reported that auxin production occurred in the shoot apical meristem (SAM) and the young leaves before it was transported toward the roots by polar movement through the stem, and auxin could improve the tolerance of plants to abiotic stress. Xu et al. (2013) also covered that the tomato 14-3-3 protein TFT4 modulated basipetal auxin transport, and the PKS5-J3 pathway for maintaining primary root elongation response to alkaline stress. Therefore, we guessed that the better root activity under saline-alkali stress, which was resulted from spraying GR24 on apple leaves, was caused by the increased IAA content in apple leaves and transport to roots response to saline-alkali stress through polar transport Zhan et al., 2018). Moreover, G-protein kinase (GK) is an important kinase in plant response to salt stress (Lian et al., 2018; Shen et al., 2019). NPK1-related protein kinase (ANP2) plays an important role in abiotic stress in rice (Ning et al., 2008). The expression levels of *MhGK* and *MhANP2* were induced by salinity-alkalinity stress but inhibited by GR24 treatment (Figure 8). Therefore, these two kinase genes would participate in response to salinity-alkalinity and GR24 treatment. Mitogen-activated protein kinase (MAPK) pathway reportedly participates in the signaling pathway of salt stress in plants, such as peppermint (*Mentha piperita*) and cucumber (*Cucumis sativus*) (Xu et al., 2011; Li et al., 2016). In the present study, the transcript level of *MhMAPKKK* showed almost the opposite trend as that of K^+^-outward rectifier gene *MhSKOR*. This finding indicates that the potential mechanisms of post-translational modification play an important role in mediating the signaling pathway of salinity-alkalinity stress. In addition, MYB, NAC and ERF transcription factors serve as connecting links between the upstream signal and the expression of functional genes under salt stress (Blumwald, 2003; Ju et al., 2020). Here, we found that these genes might participate in the GR24 signaling transduction pathway under salinity-alkalinity in apple.

Strigolactone (SL) signaling pathway enzymes include SL receptor D14, transcriptional repressor protein D53/MXL6/7/8, and F-box protein D3/MAX2 (Yao et al., 2016; Shabek et al., 2018). The transcript levels of the four SL signal transduction pathway genes, namely, *MhD14-1*, *MhD14-3*, *MhMAX2*, and *MhD53* were substantially induced by exogenous GR24 treatment (Figure 9). Similar results were also observed in the KCl stress of apple seedlings, in which the expression levels of *MdD14*, *MhMAX2*, and *MhD53* were induced by SL treatment in apple leaves. Furthermore, the decrease of SLs in tomato might be a systemic signal of drought stress (Visentin et al., 2016). Our results showed that the expression of four SL biosynthetic enzyme genes, namely, *MhCYP711*, *MhCCD7*, *MhCCD8*, and *MhD27* were decreased by salinity-alkalinity stress but substantially increased by exogenous GR24 treatment (Figure 9). Therefore, the expression levels of these four genes decreased might be an energy-saving strategy for apple to cope with salinity-alkalinity stress. Interestingly, the expression tendency of SL biosynthetic enzyme genes and *MhD14-1* were similar with *MhCHX15*, *MhSOS1*, and *MhAHAs* under salinity-alkalinity and GR24 treatment, and those of *MhD53* and *MhSKOR* were opposite, indicating the potential relationship between them. Overall, these ion transporters, kinases, transcription factors, and the SL biosynthesis and signal transduction pathway genes might have complicated regulation and interaction mechanisms. However, the mechanisms of SL signaling pathway under salinity-alkalinity stress require further analysis.

## Conclusion

Our study explored that strigolactones could effectively improve the tolerance on salinity-alkalinity stress in apple. Exogenous GR24 could affect ion homeostasis by regulating Na^+^/K^+^ transporter genes, eliminate ROS through enhancing the activities of SOD, POD, and CAT, regulate osmotic balance by increasing the proline content, balance root pH through secretion of organic acid, and cooperate with IAA, GA3, ZR, JA responding to saline-alkali stress (Supplementary Table 3). This work will provide theoretical basis for analyzing the mechanism of SL on salinity-alkalinity stress in apple plants.

## Data availability statement

The original contributions presented in this study are included in the article/Supplementary Material, further inquiries can be directed to the corresponding author.

## Author contributions

XZ and CW planned and designed the research. CB, CM, WL, XX, XL, DG, ZS, and YT performed the experiments, conducted the fieldwork, and analyzed the data. XZ and CM wrote the manuscript. All authors contributed to the article and approved the submitted version.

## Conflict of interest

The authors declare that the research was conducted in the absence of any commercial or financial relationships that could be construed as a potential conflict of interest.

## Publisher’s note

All claims expressed in this article are solely those of the authors and do not necessarily represent those of their affiliated organizations, or those of the publisher, the editors and the reviewers. Any product that may be evaluated in this article, or claim that may be made by its manufacturer, is not guaranteed or endorsed by the publisher.
