# Supplementary material for: Strigolactone alleviates the salinity-alkalinity stress of Malus hupehensis seedlings
Source: Front Plant Sci. 2022 Jul 22;13:901782. doi: 10.3389/fpls.2022.901782 (PMC9354494; doi:10.3389/fpls.2022.901782)
Supplement: Supplementary file 1 [file Data_Sheet_1.docx]

***Supplementary Material***

Strigolactone Alleviates the Salinity–alkalinity Stress of *Malus hupehensis* Seedlings

Changqing Ma, Chuanjie Bian, Wenjie Liu, Zhijuan Sun, Xiangli Xi, Dianming Guo, Xiaoli Liu, Yike Tian, Caihong Wang, Xiaodong Zheng

***Corresponding author: Xiaodong Zheng**: [zheng.xiao.d@163.com](mailto:zheng.xiao.d@163.com)

1. **Supplementary Figures**

**
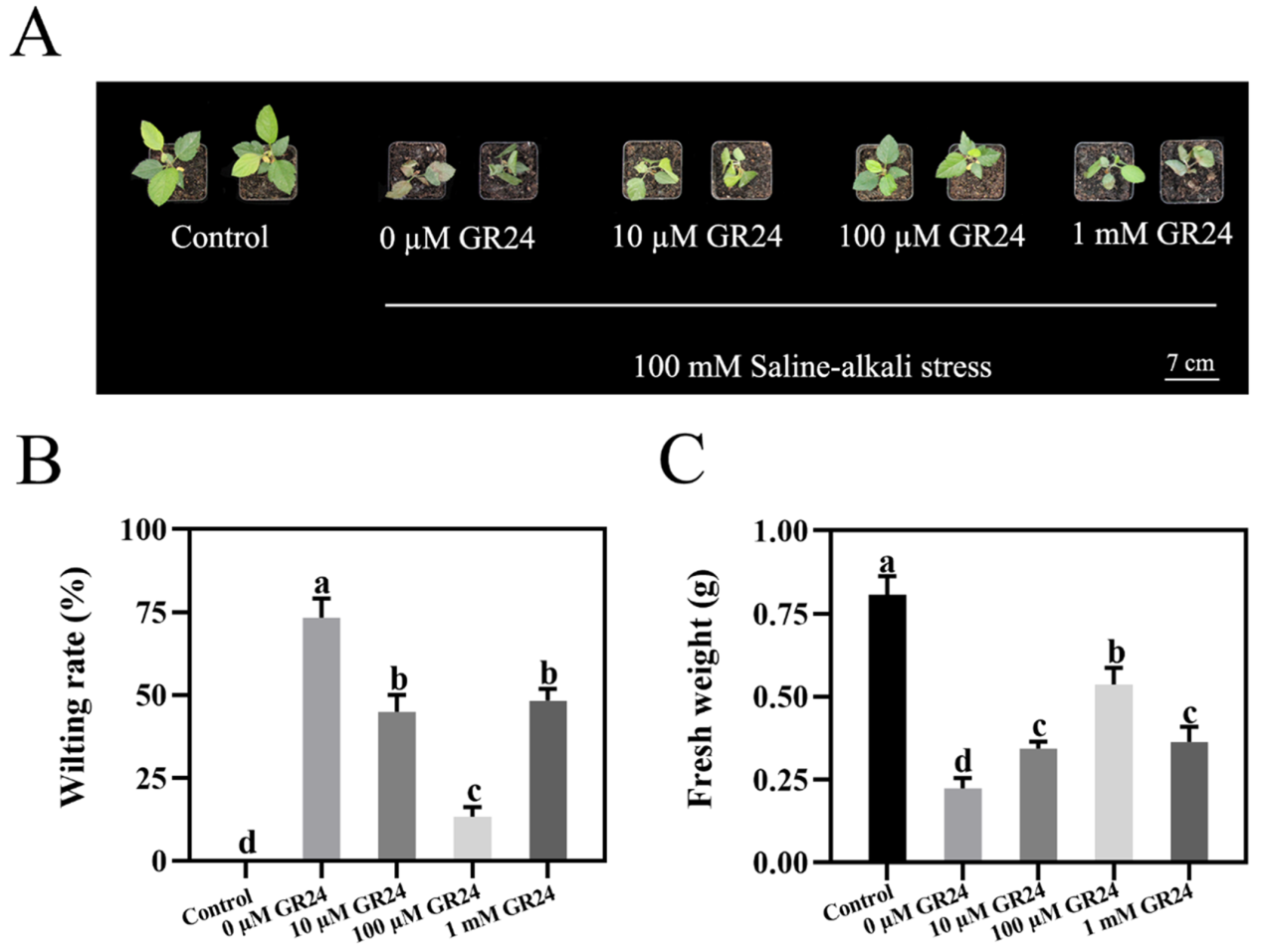
Supplementary Figure 1.** Phenotypes of *Malus* *hupehensis* seedlings treated with salinity-alkalinity stress and exogenous different concentrations of GR24 (0 μM, 10 μM, 100 μM, and 1 mM) after salinity-alkalinity stress for 15 days **(A)**. The effect under different concentrations of GR24 on wilting rate **(B)** and fresh weight **(C)** of apple seedlings after salinity-alkalinity stress for 15 days. The data represent the mean ± SD of three biological replicates. Different lowercase letters indicate significant differences, according to Fisher’s LSD (*P* <0.05).

1. **Supplementary Tables**

**Table S1** The primers used for qRT-PCR

| Gene name | Forward primer（5´-3´） | Reverse primer（5´-3´） |
| --- | --- | --- |
| *MhCHX15* | CCTCTTGGTACAGCATTGATAAAAA | GTTTGAACTTAATTTTGCAGCACA |
| *MhSOS1* | TACATCATTTCTGGTATATCTTGTG | CAAGATGAAAATTAAGGTATTAGCA |
| *MhCAX5* | ACCAGTCTCACTCTTTGTGGCG | GAATATAATTGGTGGGAGGAGATAT |
| *MhSKOR* | CATCCTGACAACTGGTGGTATCG | AAGTACCTCAGAGCAATCCGTTT |
| *MhNHX1* | TTCTGCGTGAACTTTAGACCCT | AAGACTGAGATTTCCTTTCAAGC |
| *MhNHX2* | CCACATTGATTCCAGTATTGCTT | CTCTTGAACTCTCCGTCACATTG |
| *MhGPX6* | TTCCGAGAGTAAATCAATCCACG | AGGCAAACTCTACAATCTCGTCA |
| *MhPER65* | GGCATTCTATTCCCATTCCCTT | GAGTTGGAAGCGATGAGGAGG |
| *MhpoxN1* | GCTCCTCCAAATCATTGTTACTG | AAGAAGGACAGAAGCATCACAAC |
| *MhSOD* | TGTAAATTGGCAATGATAGGAGGCC | CGTCCGGTGGAAAGAGAGAGAA |
| *MhPOD* | CGTCGTGACTGTGGCACAAA | AACGACTACCCATCTCCCCG |
| *MhCAT* | TTTCCCTGTCTTCTTCATCCGTGA | TGTGAGACTTTGGGTTGGGCT |
| *MhAHA1* | CCAGAGAAAACAAAAGAGAGTC | TTCACATTCACACCGAGATTG |
| *MhAHA3* | TATTCTCTGCCTGTTGGTCATC | GCTCACTCCATTTTCCATCTCT |
| *MhAHA9* | TTCATGGTCTTCAGCCACCT | TCACAACTGACTCGACGTGA |
| *MhANP2* | AGCATTTAGCCATTCAGGCATAC | GCAAAAAAGACGAGCTGGAGAG |
| *MhMAPKKK* | GCTACATGGGACATGACATTACTAG | CCAAGATGTTCCTGCCTTTTAT |
| *MhGK* | ACTCTGTTTGTGAAGGTTCGGTCTA | ACAAGATTACTGAGCTTTCCAGTGT |
| *MhMYB39* | AATGGGAATTGATCCTGTCACC | TGAGGTTGGATTTGGGGGTT |
| *MhERF109* | ATGCCCTTCCATGCGAATAG | CTAGTTCATGGACAACCATGCC |
| *MhNAC56* | ATGGAGTGCACCGACTCGTC | CTATCCCAAATTGGACTCAGAATAC |
| *MhD14-1* | TTTTCATTTCAGGTTTGATGTTTTG | TTGGAAGTCTCAAAAACACGAAA |
| *MhD14-3* | AGTGAGAGACTGTAAAGTTGAAAGA | CCTGAGAAAACTTGGAAAAAGACAT |
| *MhMAX2* | TGTGTTGAACTCTGTTGAACTACTG | GAGGACTATCATCTTTTTCAGCAC |
| *MhD53* | CTATGGAAGTTACTTAAAGTTCGTGGA | CATTGATACGAGCTACTTATTGGGAG |
| *MhCYP711* | ATCAGATGCCAACTGCTTATGAT | TTGGTAGAAGATAGCCTCCTATTTC |
| *MhCCD7* | TTCCCAAACTAATATTCCCCTCAC | TTCCCAAACTAATATTCCCCTCAC |
| *MhCCD8* | ATAACATTTTCAGGCACTACTGGAA | TTTTTGGGGGTGGATGAGTTAT |
| *MhD27* | GAAGGGAAAAGAGTTTACAAAGAT | GGAAAATACTGGCTTTGGGAA |
| *MhActin* | CTTCAATGTGCCTGCCATGTAT | AATTTCCCGTTCAGCAGTAGTG |

**Table S2** Abbreviations

| Abbreviations | Full name |
| --- | --- |
| CAT | Catalase |
| GA3 | Gibberellin3 |
| SOD | Super Oxide Dimutese |
| POD | Peroxidase |
| IAA | Auxin |
| JA | Jasmonic acid |
| MDA | Malondialdehyde |
| qPCR | Quantitative Real-time PCR |
| RNA | Ribonuclease acid |
| ROS | Reactive oxygen species |
| RT-PCR | Reverse transcription PCR |
| SL | [Strigolactones](https://www.sciencedirect.com/topics/agricultural-and-biological-sciences/strigolactone) |
| SPAD | The relative chlorophyll content |
| TTC | 2,3,5-triphenyltetrazolium chloride |
| ZR | Zeatin riboside |

**Table S3** The correlation of physiological mechanism and molecular mechanism between the traits

|  | Physiological mechanisms | Molecular mechanisms |
| --- | --- | --- |
| Ion homeostasis | Na^+^ content | *MhCHX15*, *MhSOS1*, *MhCAX5* |
|  | K^+^ content | *MhSKOR*, *MhNHX1*, *MhNHX2* |
| Antioxidant system | Antioxidant enzyme activities, ROS levels, and MDA content | *MhGPX6*, *MhPER65*, *MhpOXN1*, *MhSOD*, *MhPOD*, *MhCAT* |
| pH balance | Organic acid content and H^+^-ATPase enzyme activity | *MhAHA1*, *MhAHA3*, *MhAHA9* |
| Plant [hormone](javascript:;) | IAA, GA3, ZR, and JA content | *MhCYP711*, *MhCCD7*, *MhCCD8*, *MhD27* |
